# Metastatic Porocarcinoma Effectively Managed by Pembrolizumab

**DOI:** 10.7759/cureus.20004

**Published:** 2021-11-29

**Authors:** Arminder Singh, Lam Nguyen, Stephanie Everest, Mikhail Vinogradov

**Affiliations:** 1 Internal Medicine, Cape Fear Valley Medical Center, Fayetteville, USA; 2 School of Medicine, Campbell University School of Osteopathic Medicine, Lillington, USA; 3 Hematology and Oncology, Cape Fear Valley Medical Center, Fayetteville, USA

**Keywords:** immunotherapy, pembrolizumab, malignant neoplasm of eccrine glands, malignant eccrine poroma, eccrine porocarcinoma, porocarcinoma

## Abstract

Eccrine porocarcinoma (EPC) is a rare malignancy of the sweat glands. Currently, there is no standard algorithm for its presentations, diagnosis, and management. However, immunotherapy is an emerging option that may be crucial to the treatment of EPC. This report presents a case of a 79-year-old male who had a skin biopsy of an anterior scalp lesion, which revealed EPC. The patient underwent Mohs micrographic surgery to excise the tumor followed by two additional Mohs surgeries for recurrence and adjuvant radiotherapy. A follow-up positron emission tomography (PET) scan revealed yet another recurrence at the scalp as well as metastases to the left parotid gland and left submandibular lymph node. The patient was started on immunotherapy with pembrolizumab, a programmed cell death protein 1 (PD-1)/programmed death-ligand 1 (PD-L1) inhibitor, and later achieved remission. This report demonstrates the effective management of EPC using immunotherapy with pembrolizumab.

## Introduction

Eccrine porocarcinoma (EPC), or malignant eccrine poroma, is a rare skin cancer of the cutaneous intraepidermal ducts of sweat glands. Porocarcinoma accounts for less than 0.01% of all cutaneous malignancies, and it is generally seen in adults aged 50 to 80 with conflicting information regarding gender predominance [1,2]. Reports show that EPC can develop de novo or originate from a pre-existing eccrine poroma, a benign growth of the sweat glands. The lower extremities along with the head and neck region are two of the most commonly affected areas, followed by the trunk and upper extremities [3]. Due to high rate of recurrence and metastasis, porocarcinoma can pose serious consequences. It is rather challenging for clinicians to effectively manage EPC, as there are no standardized recommendations for the presentation, diagnosis, and management of porocarcinoma due to its rarity. Surgery remains the mainstay treatment for the primary porocarcinoma [1,2]. On the other hand, there is no current guideline to manage metastatic porocarcinoma, but the options include radiotherapy, chemotherapy, and electrocautery along with surgery [3].

This case report will highlight the role of pembrolizumab immunotherapy in treating patients with EPC. Pembrolizumab is a humanized monoclonal immunoglobulin G4 (IgG4) antibody typically used in the treatment of melanoma, lung cancer, Hodgkin's lymphoma, head and neck cancer, and stomach cancer. The drug selectively binds and blocks the programmed cell death protein 1 (PD-1) receptor on the cell surface, preventing the binding and activation of immune-suppressing ligands programmed death-ligand 1 (PD-L1). PD-1 is considered an immune system checkpoint. The cancer cells use PD ligands to bind to PD surface receptors on the T cell to prevent T cells from killing other cells, including cancer cells. Therefore, the inactivation of the PD-1 pathway via pembrolizumab allows activation of the T-cell-mediated immune response against tumor cells [4].

## Case presentation

**Figure 1:**
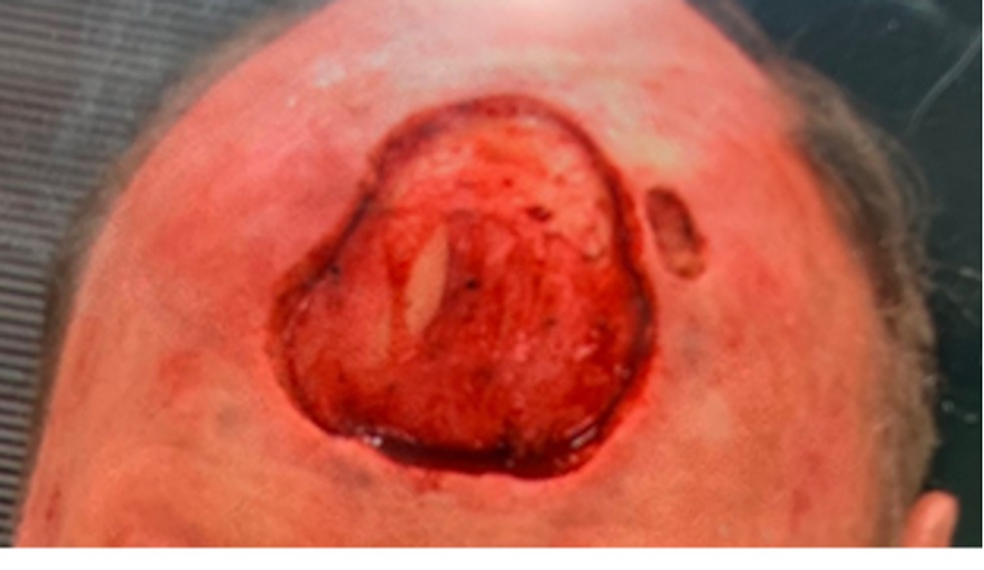
Patient 's anterior scalp during Mohs surgery

**Figure 2:**
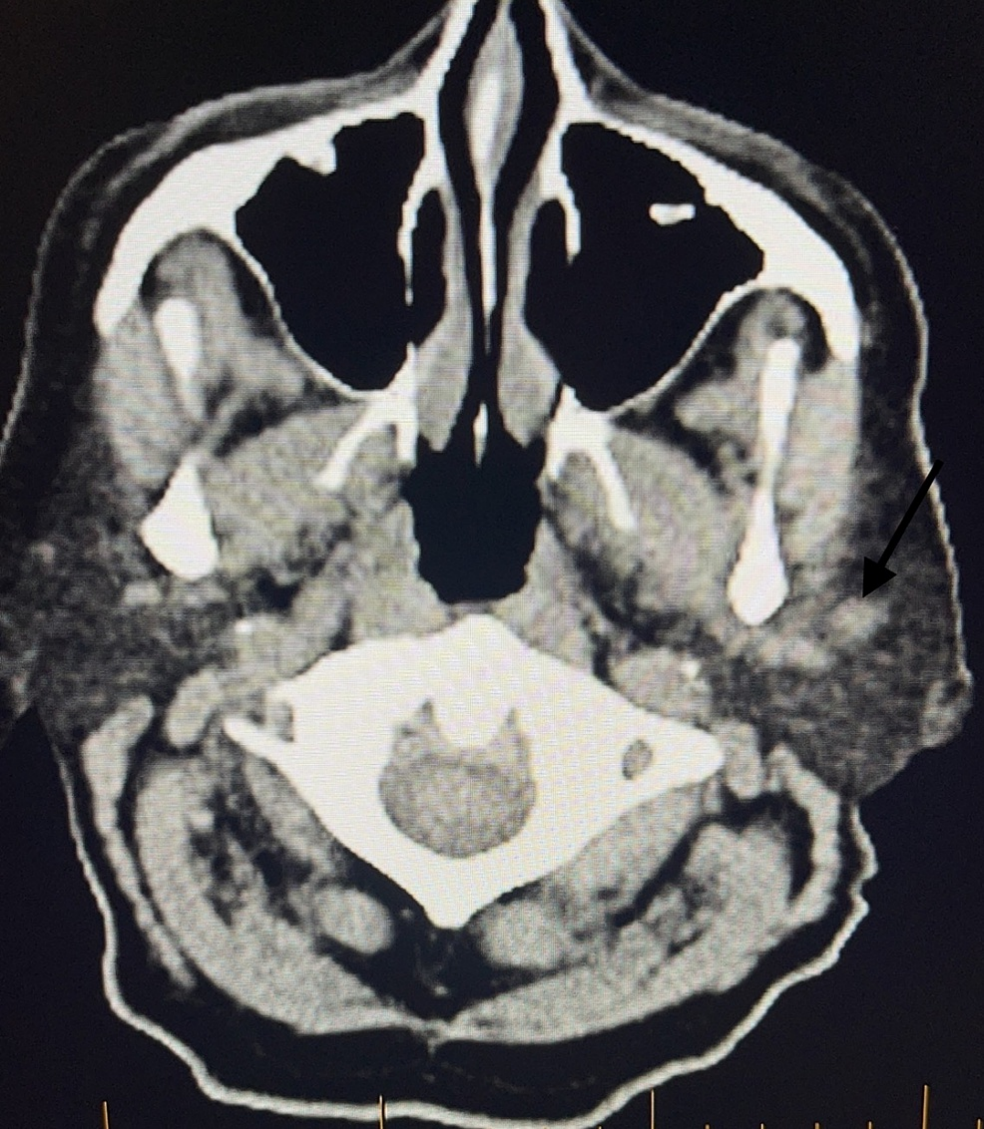
PET scan representing the first lesion near parotid gland (black arrow) PET, positron emission tomography

**Figure 3:**
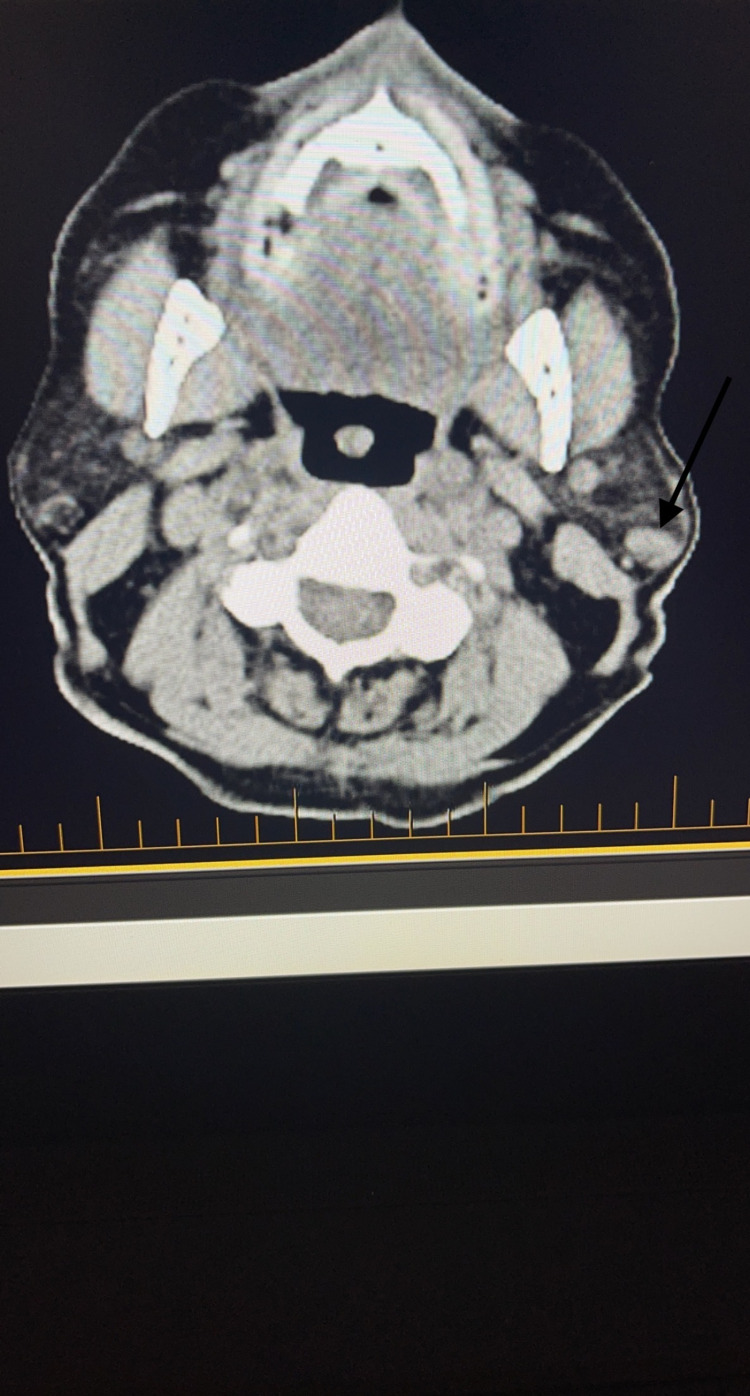
PET scan representing the second lesion near parotid gland (black arrow) PET, positron emission tomography

A 79-year-old male with a past medical history of coronary artery disease, hypertension, and stage IV chronic kidney disease stage presented with a skin lesion on his anterior scalp. Similar to many other patients with EPC, this patient’s lesion was initially thought to be due to a skin cancer with higher prevalence like squamous cell carcinoma or basal cell carcinoma. Ultimately, the diagnosis of porocarcinoma was made following a skin biopsy and pathology confirmation. Skin biopsy revealed that the tumor showed positive immunoreactivity for EMA, CK34 beta, P63, CK AE1/AE3, CEA, Ki67, D240, and MITF, and negative immunoreactivity to s100, CK7, and CK20. Skin biopsy also demonstrated porocarcinoma negative for PD-L1 by ZR3 immunostain, inflammation positive for PD-L1 by ZR3 immunostain, and PD-1-positive lymphocytes present and tumor positive for LAG3. Mohs micrographic surgery (MMS) was performed to remove his original tumor (Figure 1). The patient unfortunately had two recurrences of his EPC, and both of these recurrent lesions were removed by MMS as well as adjuvant radiotherapy. After the third Mohs surgery, which was 11 months after the initial diagnosis of his porocarcinoma, the patient had a positron emission tomography (PET) scan that revealed two new hypermetabolic lesions near the left parotid gland, a new right paramedian frontal scalp thickening with increased metabolic activity consistent with recurrence, and an enlarged left submandibular lymph node with increased metabolic activity (Figures 2, 3). 

**Figure 4:**
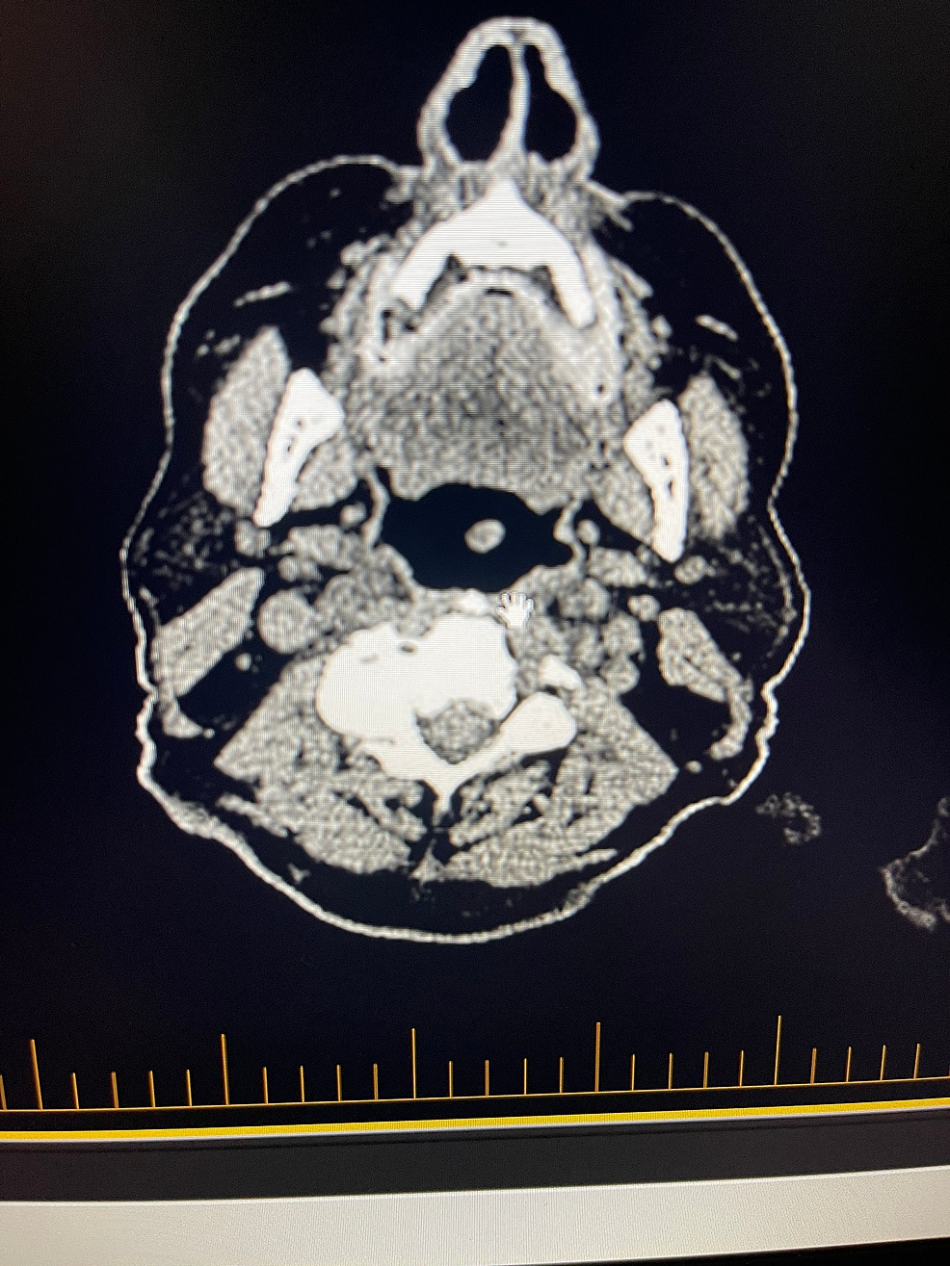
PET scan demonstrating resolution of lesion seen in Figure 2 after 9 months of immunotherapy with pembrolizumab PET, positron emission tomography

**Figure 5:**
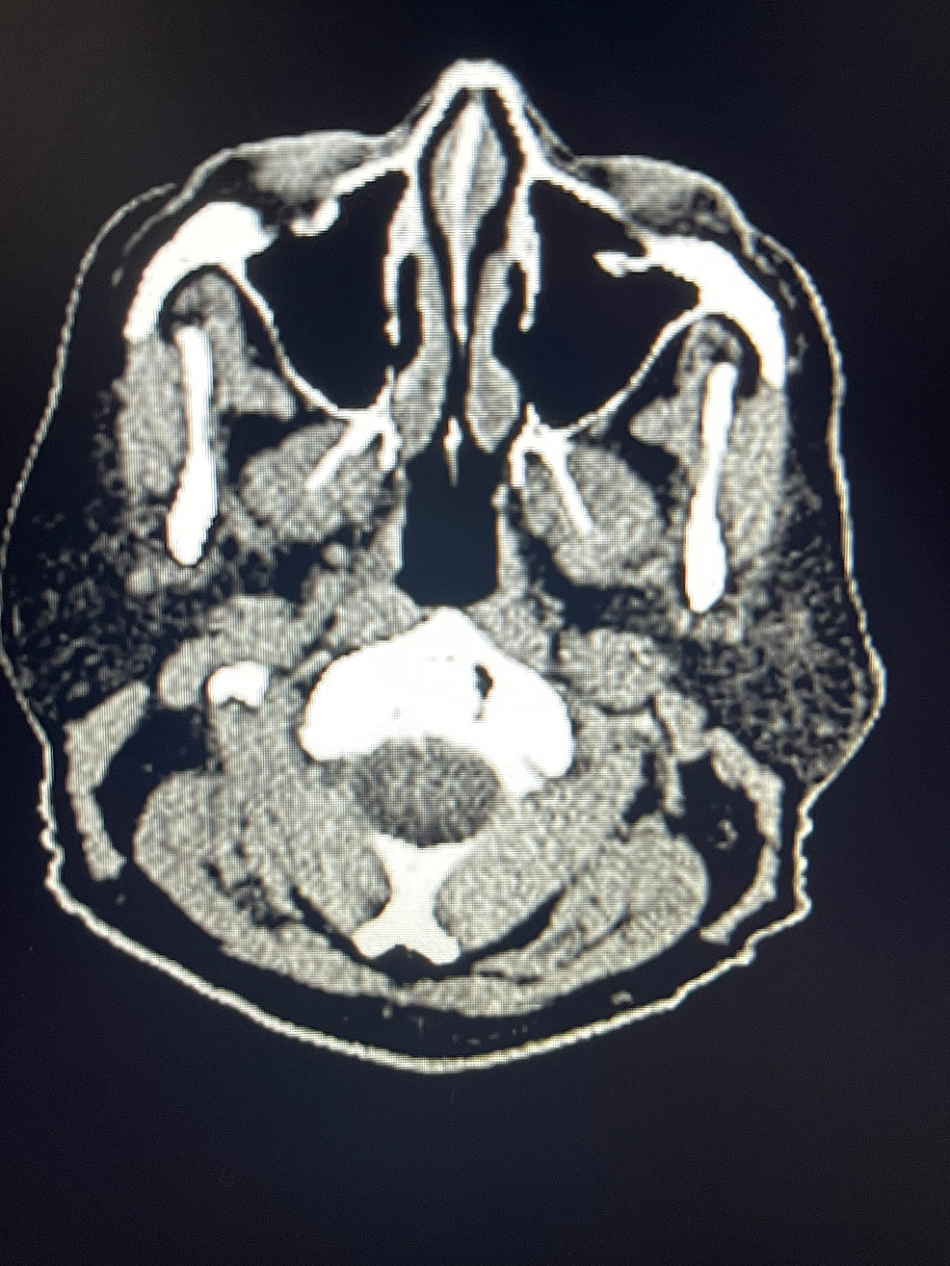
PET scan demonstrating resolution of lesion seen in Figure 3 after 9 months of immunotherapy with pembrolizumab PET, positron emission tomography

**Figure 6:**
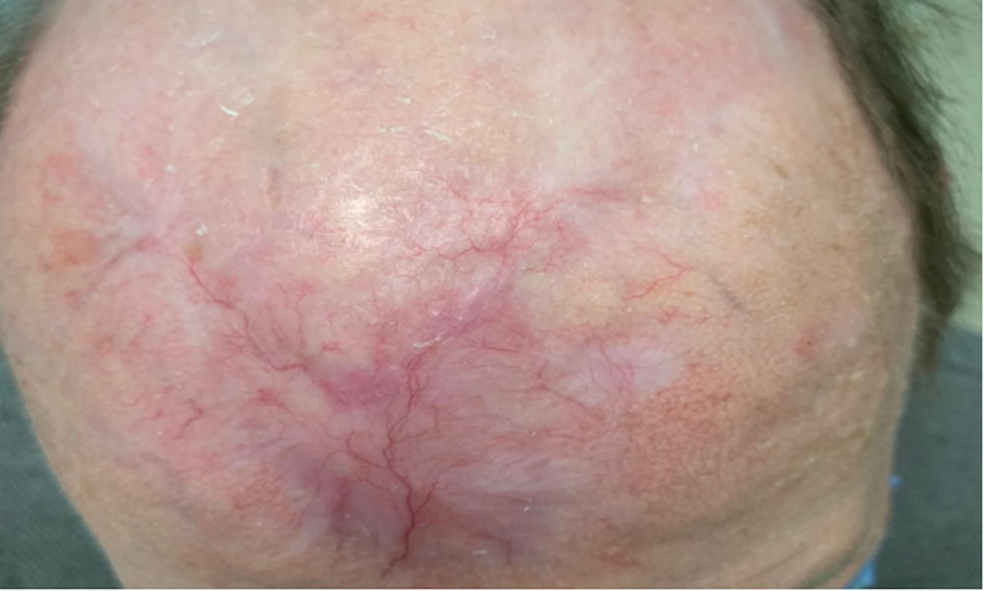
Patient’s anterior scalp after three Mohs micrographic surgeries with adjuvant radiotherapy and 11 months of receiving pembrolizumab

Immunotherapy of pembrolizumab was started using a melanoma protocol for the above-mentioned metastatic tumors. Nine months after the initiation of his immunotherapy, the patient underwent a second PET scan, which showed resolution of previously documented hypermetabolic skin thickening of the frontal skull, significant improvement of hypermetabolic masses within the bilateral parotid glands, and interval progression of nodal disease in the left submandibular region (Figures 4, 5). A repeat PET scan was performed five months after the second scan, and the results demonstrated interval resolution of previously seen hypermetabolic lymphadenopathy within the left neck and no evidence of metastatic disease within the chest. It has been 27 months since the diagnosis of metastatic disease and initiation of immunotherapy, and the patient is in remission and currently following up with his oncologist to continue immunotherapy with pembrolizumab (Figure 6). 

 

## Discussion

EPC is a rare but aggressive malignancy of the eccrine sweat glands representing around 0.01% of all malignant cutaneous neoplasms. Morphologically, EPC can present as a nodule, ulcerated lesion, or plaque that is painful or painless with a wide spectrum of colors [2]. Furthermore, these lesions can have various characteristics including spontaneous bleeding, ulceration, itching, or sudden growth. These make it challenging to diagnose porocarcinoma using clinical presentations, as it is difficult to differentiate EPC from other cutaneous lesions such as squamous cell carcinoma, basal cell carcinoma, amelanotic melanoma, seborrheic keratosis, or verruca vulgaris. Therefore, histopathology with immunohistochemistry remains the gold standard in diagnosing porocarcinoma [2,3]. A 2017 meta-analysis of 453 cases reveals that metastasis of EPC is found in 31% of cases, reflecting the fact that clinicians encounter metastasis rather commonly among patients with porocarcinoma. The same analysis shows that regional lymph nodes are the most common site of metastasis at 53%. Other frequent metastatic sites include brain, bone, lung, liver, and skin [3].

Due to the disease rarity, there is no established algorithm for management. Surgical treatments including wide local excision and MMS have been shown to be the absolute necessity for EPC curative therapy [2]. Patients who were treated using these surgical techniques had significantly superior outcomes with MMS providing better esthetic results. For patients with metastatic porocarcinoma, surgery remains to be the first-choice option [1,2]. Additionally, other alternatives have shown promising results including chemotherapy, radiotherapy, and electro-chemotherapy. There are multiple case reports demonstrating great clinical outcomes from different chemotherapy treatments such as maxacalcitol/imiquimod, docetaxel, or a combination of 5-fluorouracil, cisplatin, doxorubicin, vincristine, and mitomycin [1,5-7]. Another option oncologists are exploring is immunotherapy. At the time of writing this article, we found one case report from 2019 documenting the use of pembrolizumab in EPC, in which a patient achieved remission with pembrolizumab after surgical excision, radiotherapy, and 12 cycles of chemotherapy had failed to stop the disease progression [8].

In our patient, the diagnosis was made by histopathology of his skin biopsy. Mohs surgery was attempted and it successfully treated his primary EPC. Repeat MMS and adjuvant radiotherapy were also performed to treat the recurrences of the patient's cancer. However, the patient was unfortunately found to have metastases to the submandibular lymph node and left parotid gland, which had been effectively managed with immunotherapy of pembrolizumab. The patient has been in remission for 18 months. With this, pembrolizumab can potentially become one of the alternative treatment options or even primary therapy for metastatic porocarcinoma. However, further research is required to prove its effectiveness and safety across the EPC patient population. As of now, pembrolizumab may be considered for palliative care and part of a multistep treatment for patients with EPC. 

## Conclusions

Our patient had a great response to the immunotherapy treatment with current remission. From this case report, we conclude that pembrolizumab can serve as an effective palliative therapy or even a definitive treatment of metastatic porocarcinoma. Yet, further research is needed to prove its efficacy and safety in treating EPC.
